# Inhibition of Ferroptosis Attenuates Neuron Damage and Improves Cognitive Impairment in Mice Surviving Severe Hypothermia

**DOI:** 10.3390/ijms26114965

**Published:** 2025-05-22

**Authors:** Wei-Xuan Li, Xue-Tong Dong, Fu Zhang, Jun-Yan Wang, Chao-Long Lu, Zhao-Qi Zhou, Jia-Yi Gu, Song-Jun Wang

**Affiliations:** 1 Undergraduate Program of College of Forensic Medicine, Hebei Medical University, No. 361 Zhong Shan Road, Shijiazhuang 050017, China; 2 Hebei Key Laboratory of Forensic Medicine, Collaborative Innovation Center of Forensic Medical Molecular Identification, Research Unit of Digestive Tract Microecosystem Pharmacology and Toxicology, Chinese Academy of Medical Sciences, College of Forensic Medicine, Hebei Medical University, No. 361 Zhong Shan Road, Shijiazhuang 050017, China; 3 Forensic Pathology Laboratory, Guangdong Public Security Department, No. 97 Huanghua Road, Guangzhou 510050, China; coronerzhang@outlook.com

**Keywords:** ferroptosis, hypothermia, cognitive impairment, cerebral cortical, nerve cells

## Abstract

Survivors of severe hypothermia frequently exhibit cognitive impairments. However, the underlying mechanisms remain inadequately understood. In order to reveal the scientific problem of cognitive dysfunction caused by severe hypothermia, providing an experimental basis for clinical treatment, this study utilized animal models and combined cognitive behavioral, morphological, and molecular biological experiments. The results showed that severe hypothermia leads to an accumulation of iron ions in the cerebral cortex tissue exceeding 70%, while increased Acyl-coenzyme A synthetase long-chain family member 4 (ACSL4) expression enhances sensitivity to ferroptosis. This process results in a nearly 50% decrease in glutathione (GSH) expression and over 50% degradation of glutathione peroxidase 4 (GPX4), leading to GPX4 deactivation and increased lipid peroxidation, which in turn nearly doubles the levels of oxidative products such as MDA and 4NHE. Notably, ferroptosis inhibition using Ferrostatin-1 (Fer-1) effectively mitigates the degenerative death of cerebral cortical neurons induced by severe hypothermia, significantly improving the associated cognitive deficits. These findings suggest that severe hypothermia may induce ferroptosis in cortical neurons through the Nrf2/SLC7A11/GSH/GPX4 signaling axis. Targeted inhibition of ferroptosis has the potential to be a promising therapeutic direction for the prevention and treatment of cognitive impairment caused by severe hypothermia.

## 1. Introduction

The cold climate poses a significant threat to human health and safety [1]. In regions and seasons characterized by extreme cold, the intensity, frequency, and severity of cold weather events are increasingly exacerbated by ongoing climate change [2]. As a result, cold exposure has become a severe risk for individuals engaged in field exploration, leading to a marked rise in cases of frostbite and freezing injuries [3,4]. Clinically, patients who experience hypothermic coma often suffer from long-term cognitive impairment, though the underlying mechanisms remain poorly understood. Previous studies have shown that severe hypothermia induces metabolic disturbances in cerebral cortical neurons, leading to significant alterations in genes associated with ferroptosis, thereby promoting ferroptosis-related damage [5]. However, the role of ferroptosis in cognitive impairment caused by severe hypothermia remains unexplored.

Ferroptosis is characterized by intracellular iron accumulation, failure of the antioxidant defense system, and lipid peroxidation. The SLC7A11/GSH/GPX4 axis represents a critical GSH-dependent defense mechanism against ferroptosis and is one of the most essential antioxidant pathways protecting cells from ferroptotic damage [6,7]. The amino acid reverse transporter complex consists of the light chain subunit, Solute Carrier Family 7 Member 11 (SLC7A11), and the heavy chain subunit, Solute Carrier Family 3 Member 2 (SLC3A2), which together facilitate the synthesis of glutathione (GSH). GSH is the most abundant reductant in mammalian cells, and its biosynthesis, along with glutathione peroxidase 4 (GPX4), mediates the reduction of phospholipid hydroperoxides (PLOOH) to corresponding phospholipid alcohols (PLOH). The inhibition of GPX4 prolongs the presence of phospholipid hydroperoxides, triggering a Fenton reaction that exacerbates the accumulation of PLOOHs, a key feature of ferroptosis. Lipid peroxidation produces initial lipid hydroperoxides and reactive aldehydes, such as malondialdehyde (MDA) and 4-hydroxynonenal (4-HNE), which increase during ferroptosis. The buildup of PLOOHs leads to rapid and irreversible damage to cell membranes, culminating in cell death [8,9]. Nuclear factor E2-related factor 2 (Nrf2) is a central regulator of endogenous antioxidant pathways, crucial for maintaining cellular redox balance. Nrf2 modulates the expression of key proteins involved in iron metabolism, including the light and heavy chains of ferritin, and regulates SLC7A11 of the GSH antioxidant system [10,11]. Previous studies have found that ferroptosis mediates the process of cold-induced damage to the heart, kidney, and liver [1,12,13]. Ferroptosis has been implicated in cognitive impairments associated with neurodegenerative diseases [14,15], hypoxic–ischemic brain injury [16], and traumatic brain injury [17]. Therefore, previous studies suggest that ferroptosis may mediate cognitive dysfunction caused by severe hypothermia.

In this study, a mouse model of survival after severe hypothermic coma was established [5]. Cognitive function was assessed using novel object recognition and the Barnes maze. Brain neuronal damage in surviving mice was examined using histopathological techniques, including HE staining and Thionin staining. Ferroptosis-related proteins, such as iron ion content in tissues, were measured to confirm ferroptosis occurrence in hypothermia-induced cognitive impairment. Additionally, the therapeutic potential of the ferroptosis-specific inhibitor Ferrostatin-1 (Fer-1) was evaluated for treating hypothermic coma. The purpose of this study is to clarify whether ferroptosis is involved in the process of neuronal damage caused by hypothermia, and whether it further causes cognitive dysfunction, so as to provide an experimental basis for the clinical treatment of patients with hypothermic coma.

## 2. Results

### 2.1. Cognitive Impairment Was Observed in Surviving Mice with Severe Hypothermia

**Figure 1:**
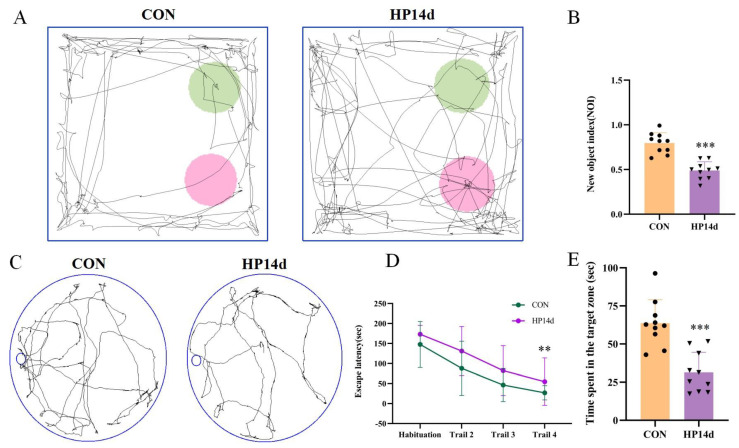
Severe hypothermia induces cognitive impairment in mice surviving severe hypothermia. (**A**) Representative images from the NOR test, with pink circles representing the familiar object and green circles representing the novel object. (**B**) Statistical analysis of the NOI in the NOR test. (**C**) Representative images from the BM experiment. (**D**) Statistical analysis of escape latency to the target box during the training phase of the BM experiment. (**E**) Statistical analysis of the time spent exploring the target object in the BM experiment. Data are presented as mean ± SEM. ** *p* < 0.01, *** *p* < 0.001 vs. the control group (*n* = 10).

The cognitive function of mice surviving severe hypothermia was assessed using the Novel Object Recognition Experiment (NOR) and Barnes Maze (BM). In the Novel Object Recognition Experiment (Figure 1A,B), the cognitive index of mice in the 14-day severe hypothermia group was significantly reduced compared to the control group (*p* < 0.001). Similarly, in the Barnes Maze (Figure 1C,E), mice in the 14-day severe hypothermia group spent significantly less time near the target box compared to the control group (*p* < 0.001). These results indicate that mice surviving severe hypothermia exhibit severe cognitive impairment.

### 2.2. Degeneration and Death of Cortical Neurons in Surviving Mice with Severe Hypothermia

**Figure 2:**
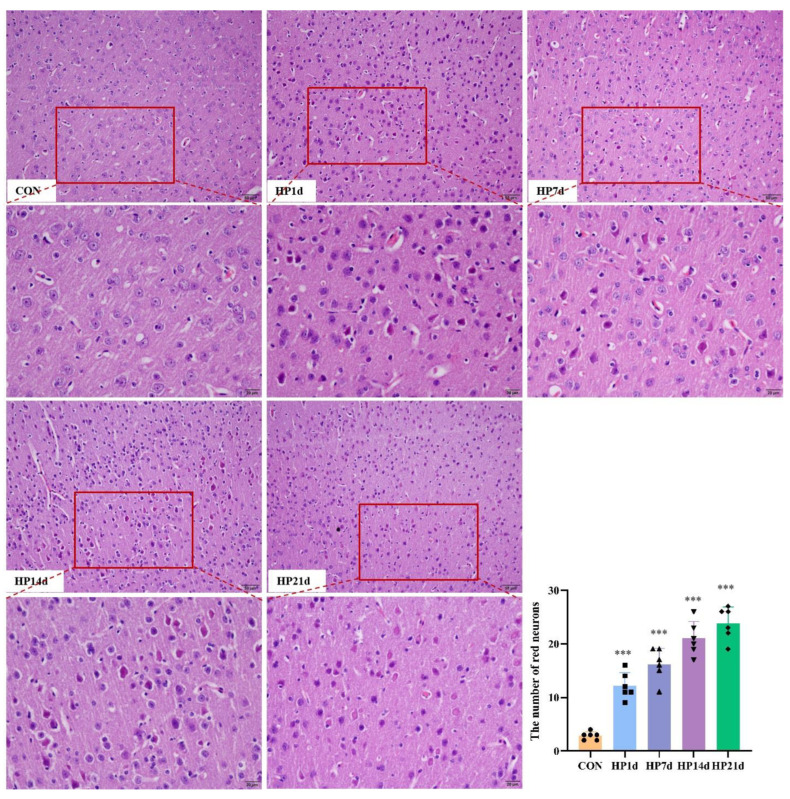
Severe hypothermia induces death of cortical neurons in mice surviving severe hypothermia. Representative HE staining images of the cerebral cortex, with neuronal death manifested as red neurons (bars = 50 μm). The enlarged image inside the red box is shown directly below (bars = 20 μm), With the survival time extended, the number of red neurons is progressively increased. Quantitative analysis of the number of red neurons in the cerebral cortex. Data are presented as mean ± SEM; *** *p* < 0.001 vs. the control group (*n* = 6).

**Figure 3:**
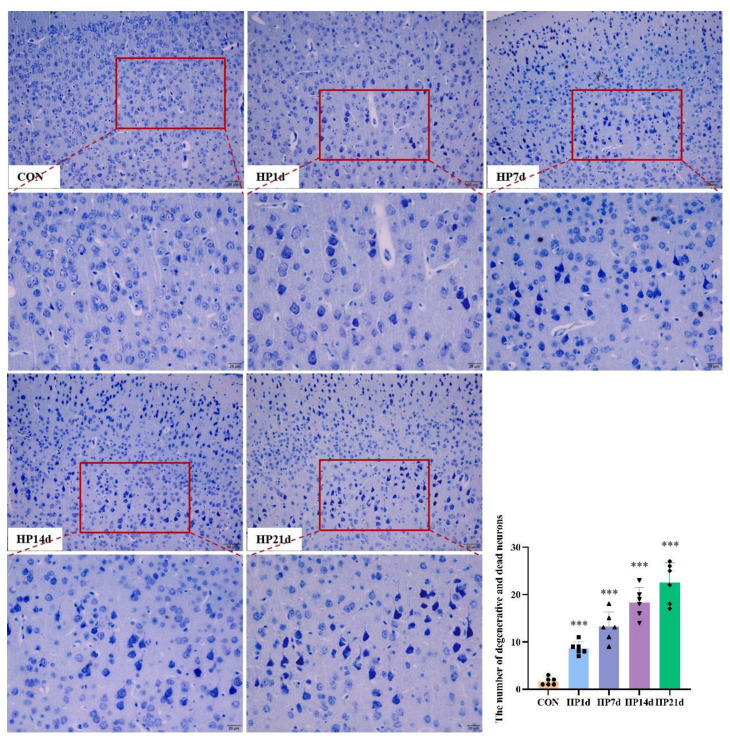
Severe hypothermia induces the degeneration of cortical neurons in mice surviving severe hypothermia. Representative Thionin staining images of the cerebral cortex (Bars = 50 μm). The enlarged image inside the red box is shown directly below (Bars = 20 μm). With the survival time extended, Nissl bodies were not clear; neurophagy and pyknotic neurons were visible. Quantitative analysis of the number of Thionin-positive cells in the cerebral cortex. Data are presented as mean ± SEM; *** *p* < 0.001 vs. the control group (*n* = 6).

Cognitive function is the result of complex interactions among multiple forebrain structures (e.g., cortex, basal ganglia, basal forebrain, dorsal thalamus), with the cerebral cortex playing a dominant role. The cerebral cortex is considered the seat of intelligence and cognitive processes in mammals. The number of neurons, particularly cortical neurons, is a critical determinant of cognitive function. In this study, HE and Thionin staining were employed to examine neuron degeneration and death morphologically. The results showed that neuron degeneration and death primarily occurred in the cerebral cortex, particularly in the parietal lobe, in mice surviving severe hypothermia (Figure 2 and Figure 3). The number of degenerated neurons in the cerebral cortex of mice surviving severe hypothermia was significantly higher than that in the control group (*p* < 0.001), with an increasing trend in neuron degeneration and death as the survival time extended. These results indicate that neuronal degeneration and death are evident in the cerebral cortex of surviving mice with severe hypothermia.

### 2.3. Ferroptosis Occurs in the Cerebral Cortex of Mice Surviving Severe Hypothermia

#### 2.3.1. Ferrous Ion Accumulation in the Cerebral Cortex of Mice Surviving Severe Hypothermia

**Figure 4:**
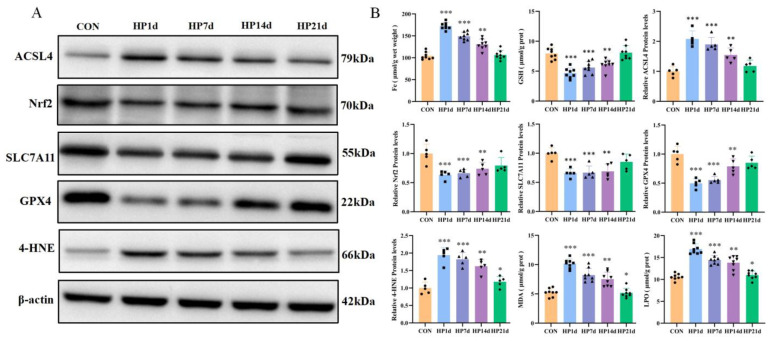
Severe hypothermia induces ferroptosis in the cerebral cortex of mice surviving severe hypothermia. (**A**) Expression of ACSL4, Nrf2, SLC7A11, GPX4, and 4-HNE in the cerebral cortex, assessed by Western blotting analysis. (**B**) Quantification of protein levels from (**A**), as well as levels of GSH, MDA, LPO, and iron content in the cerebral cortex of mice. Data are presented as mean ± SEM; * *p* < 0.05, ** *p* < 0.01, *** *p* < 0.001 vs. the control group (*n* = 5).

Ferrous ion accumulation plays a pivotal role in initiating the ferroptosis pathway [18]. The results revealed that the ferrous ion content in the cerebral cortex of mice surviving 1, 7, and 14 days of severe hypothermia was significantly higher than that of the control group (*p* < 0.01). However, no statistically significant difference in ferrous ion content was observed in the cerebral cortex of mice surviving 21 days of severe hypothermia compared to the control group (*p* > 0.05) (Figure 4B). These results suggest that ferrous ion content accumulation in the cerebral cortex primarily occurs in the early stages of survival following severe hypothermia, with iron metabolism gradually returning to normal as survival time increases.

#### 2.3.2. Pro-Ferroptosis Proteins Were Activated in the Cerebral Cortex of Mice Surviving Hypothermia Coma

ACSL4 is a key enzyme in the metabolism of polyunsaturated fatty acids (PUFAs). The upregulation of ACSL4 increases the content of polyunsaturated fatty acids in phospholipids, enhancing the sensitivity to oxidation reactions. ACSL4 has been shown to be a critical mediator in the pro-ferroptosis cascade [19]. The results revealed that the levels of ACSL4 in the cerebral cortex of mice surviving 1, 7, and 14 days of severe hypothermia were significantly higher than those in the control group (*p* < 0.01). However, no significant difference was observed in the ACSL4 levels in the cerebral cortex of mice surviving 21 days of hypothermia compared to the control group (*p* > 0.05) (Figure 4). These results suggest that the pro-ferroptosis protein ACSL4 is primarily elevated in the early stages of survival following severe hypothermic coma.

#### 2.3.3. Failure of the Antioxidant System in the Cerebral Cortex of Mice Surviving Hypothermia Coma

The depletion of antioxidant systems such as GSH, SLC7A11, and glutathione peroxidase 4 (GPX4) is a key step in triggering ferroptosis [20,21]. Nrf2 is a critical transcriptional regulator of anti-ferroptosis genes. The results indicated that the levels of GSH, SLC7A11, GPX4, and Nrf2 in the cerebral cortex of mice surviving severe hypothermia for 1, 7, and 14 days were significantly lower than those in the control group (*p* < 0.01). No significant differences were found in the levels of GSH, SLC7A11, and GPX4 in the cerebral cortex of mice surviving 21 days of hypothermia compared to the control group (*p* > 0.05) (Figure 4). These results suggest that the antioxidant system in the cerebral cortex is severely impaired in the early stages of survival following hypothermic coma, with a gradual recovery of antioxidant function as survival time increases.

#### 2.3.4. Increased Products of Lipid Peroxidation in the Cerebral Cortex of Surviving with Hypothermic Coma

The products of lipid peroxidation, including MDA, LPO, and 4-HNE, contribute to ferroptosis by exacerbating membrane damage and other mechanisms. The results demonstrated that the levels of MDA and 4-HNE in the cerebral cortex of mice surviving 1, 7, 14, and 21 days of severe hypothermia were significantly higher than those in the control group (*p* < 0.01) (Figure 4). These results indicate that severe hypothermia induces lipid peroxidation in the cerebral cortex of surviving mice.

### 2.4. Ferroptosis Inhibitors Mitigate Nerve Damage in the Cerebral Cortex in Mice Surviving Severe Hypothermia

#### 2.4.1. Ferroptosis Inhibitor Can Reduce the Expression of Ferroptosis-Related Proteins in Mice Surviving Severe Hypothermia

**Figure 5:**
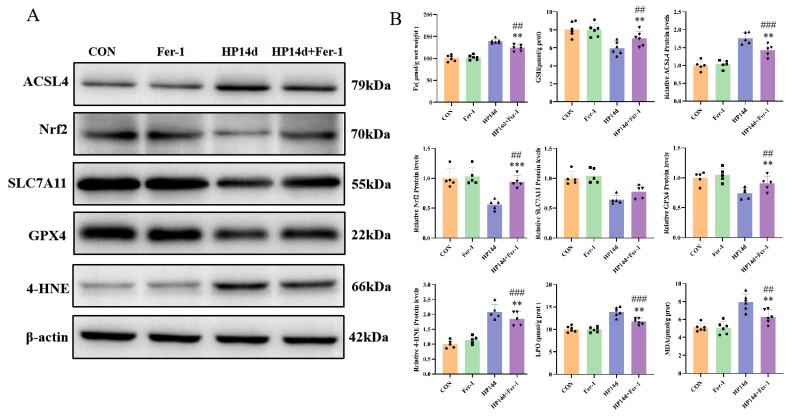
Fer-1 decreases the expression of ferroptosis-related proteins in the cerebral cortex of mice surviving severe hypothermia. (**A**) Expression levels of ACSL4, Nrf2, SLC7A11, GPX4, and 4-HNE in the cerebral cortex, assessed by Western blotting analysis. (**B**) Quantification of the protein levels from (**A**), along with the levels of MDA, GSH, LPO, and iron content in the cerebral cortex of mice. Data are presented as mean ± SEM; ** *p* < 0.01, *** *p* < 0.001 vs. the HP14d group, ## *p* < 0.01, ### *p* < 0.001 vs. the control group (*n* = 5).

Fer-1 inhibits ferroptosis primarily by regulating oxidative stress and iron ion metabolism in cells. As a highly effective and specific ferroptosis inhibitor, Fer-1 has been widely utilized in research [22]. In this study (Figure 5), compared to the 14-day severe hypothermia survival group, the Fer-1 treatment group exhibited a significant reduction in iron content in the cerebral cortex (*p* < 0.01), a notable decrease in the ferroptosis-promoting enzyme ACLS4 (*p* < 0.01), and a significant increase in antioxidant substances such as GSH, SLC7A11, GPX4, and Nrf2 (*p* < 0.01). Additionally, the levels of lipid peroxidation markers MDA and 4-HNE were significantly reduced (*p* < 0.01). These results indicate that Fer-1 effectively alleviates iron accumulation in the cerebral cortex of mice surviving severe hypothermia, enhances the antioxidant system’s capacity, increases tolerance to ferroptosis, and reduces lipid peroxide accumulation.

#### 2.4.2. Ferroptosis Inhibitor (Fer-1) Alleviated Neuronal Degeneration and Death in Mice Surviving Severe Hypothermia

**Figure 6:**
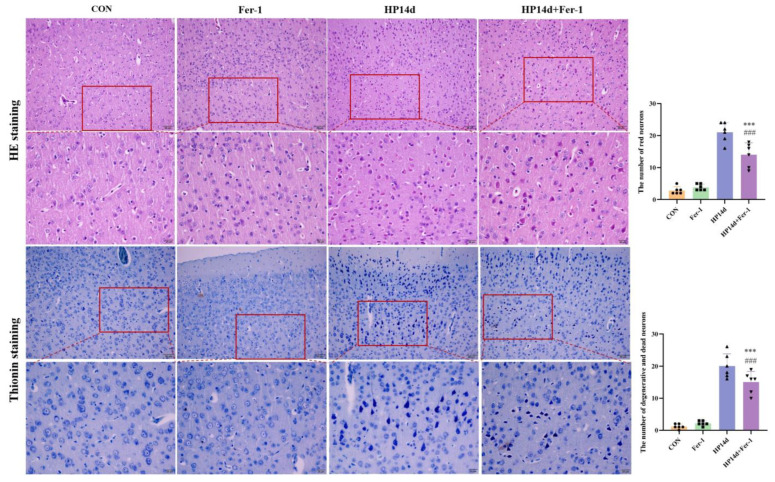
Fer-1 alleviates hypothermia-induced neuronal damage. Representative HE-stained images of the cerebral cortex (Bars = 50 μm). The enlarged image inside the red box is shown directly below (Bars = 20 μm). The results showed that Fer-1 can effectively improve the damage of cortical neurons in surviving mice with severe hypothermia. Data are presented as mean ± SEM; *** *p* < 0.001 vs. the HP14d group, ### *p* < 0.001 vs. the control group (*n* = 6).

Histological analysis using HE and Thionin staining (Figure 6) revealed that the Fer-1 treatment group showed a significant reduction in the number of degenerated and dead neurons in the cerebral cortex compared to the 14-day survival group of severe hypothermia. These results suggest that Fer-1 effectively mitigates neuronal degeneration and death in the cerebral cortex of mice surviving severe hypothermia.

#### 2.4.3. Ferroptosis Inhibitor (Fer-1) Improved Cognitive Function in Mice Surviving Severe Hypothermia

**Figure 7:**
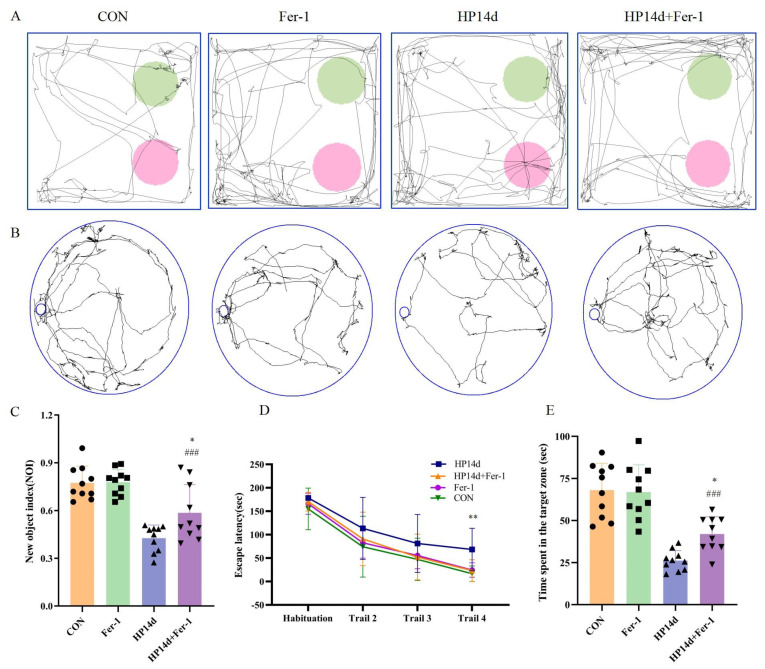
Fer-1 improves cognitive impairment induced by severe hypothermia. (**A**) Representative images from the NOR test, with pink circles representing the familiar object and green circles representing the novel object. (**B**) Representative images from the BM experiment. (**C**) Statistical analysis of the NOI in the NOR test. (**D**) Statistical analysis of the escape latency to the target box during the training phase of the BM experiment. (**E**) Statistical analysis of the time spent exploring the target object in the BM experiment. Data are presented as mean ± SEM; * *p* < 0.05, ** *p* < 0.01 vs. the HP14d group, ### *p* < 0.001 vs. the control group (*n* = 10).

Behavioral assessments showed significant improvements in cognitive function in the Fer-1 treatment group. In the NOR experiment, the cognitive index of mice in the Fer-1 treatment group was significantly higher compared to the 14-day severe hypothermia survival group (*p* < 0.05) (Figure 7C). Similarly, in the Barnes Maze, the Fer-1 treatment group spent significantly more time around the target box compared to the 14-day survival group of severe hypothermia (*p* < 0.05) (Figure 7E). These results demonstrate that Fer-1 can effectively improve cognitive function in mice surviving severe hypothermia, further supporting the role of ferroptosis in the cognitive impairment caused by severe hypothermia.

## 3. Discussion

This study demonstrates that severe hypothermia induces ferroptosis in cerebral cortex neurons, leading to neuronal degeneration and death, which subsequently causes cognitive impairment. Treatment with the ferroptosis inhibitor Fer-1 significantly alleviates cognitive dysfunction resulting from severe hypothermia. The protective effect of Fer-1 on cognitive function is attributed to its ability to reduce iron accumulation in the cerebral cortex and enhance the antioxidant capacity of the SLC7A11/GSH/GPX4 axis, thereby preventing neuronal degeneration and death in the cerebral cortex.

Neurons are essential for cognitive function, and their degeneration and death are key mechanisms underlying cognitive impairment. This process is essential for the damage to behavior and memory functions following brain injury. Previous studies have shown that exposure to low temperatures can decrease responsiveness and impair cognitive function [23,24]. Persistent hypothermia has also been linked to intellectual deficits, with synaptic plasticity playing an important role in the cognitive impairment induced by hypothermia, even leading to tau hyperphosphorylation [25]. These studies highlight the intricate relationship between hypothermia and cognitive dysfunction from various perspectives. The results of our study indicate that severe hypothermia leads to irreversible cognitive impairment through neuronal degeneration and death in the cerebral cortex. Morphological analysis revealed that the degenerative neuronal death caused by severe hypothermia predominantly affects excitatory pyramidal neurons. This may be due to excitotoxicity, wherein excessive extracellular glutamate disrupts SLC7A11-mediated cysteine import, leading to depletion of GSH and inactivation of GPX4 [26]. Furthermore, while iron levels gradually decreased with the extension of hypothermic survival time, the degeneration and death of cortical neurons continued to increase. This may be attributed to the inflammatory mechanisms triggered by ferroptosis [27].

Increasing evidence has highlighted the close relationship between ferroptosis and metabolic disorders [28]. Hypothermia itself can lead to significant metabolic disturbances in the body. For example, studies detecting metabolite differences in the internal jugular artery and vein during hypothermia have shown an increase in glutamate uptake [29]. Targeted metabolomics studies on serum and urine from hypothermia deaths have revealed elevated arginase activity, with biomarkers such as cortisol, arginine, and 3-hydroxybutyric acid being associated with cold exposure [30,31]. Both elevated glutamate and arginine levels are closely linked to increased sensitivity to ferroptosis. An increase in endogenous glutamate levels results in an elevation of cellular Ca^2+^ concentration, which activates the adenylate cyclase/protein kinase A axis, phosphorylating and inhibiting glutamine-fructose-6-phosphate transaminase (GFPT1). The decreased activity of GFPT1 leads to YAP instability, which in turn reduces the expression of its downstream target gene FTH1. This diminishes FTH1’s transcriptional replacement triggered by ferritin phagocytosis, thereby heightening the sensitivity of cells to ferroptosis [32]. Additionally, argininosuccinate synthase 1 (ASS1) facilitates the synthesis of arginine, which is a key amino acid in ferroptosis induction [33]. Arginine also plays a role in maintaining fumarate biosynthesis through the urea cycle. As a reactive α- and β-unsaturated electrophilic metabolite, fumarate can covalently bind to GSH, reducing intracellular GSH levels and ultimately promoting ferroptosis [34]. In our study, ferroptosis was found to be particularly pronounced in the early stages in mice surviving severe hypothermia, suggesting that the severity of the metabolic disturbances caused by hypothermia directly correlates with the intensity of ferroptosis. This observation aligns with extensive research indicating a strong connection between ferroptosis and various metabolic diseases, including diabetic cardiomyopathy [35] and rheumatoid arthritis [36].

Ferroptosis is a form of iron-dependent cell death characterized by the accumulation of free iron, which directly promotes excessive reactive oxygen species (ROS) production via the Fenton reaction, exacerbating oxidative damage [37]. ACSL4, an enzyme involved in fatty acid metabolism, plays a key role in ferroptosis, acting as both a specific biomarker and a driving factor. The upregulation of ACSL4 increases the incorporation of PUFAs into phospholipids, making them highly susceptible to oxidative reactions that trigger ferroptosis [8]. The hallmark of ferroptosis is the failure of the classical antioxidant system, particularly the GSH–GPX4 axis, with anti-ferroptotic mediators being suppressed through transcriptional regulation of the Nrf2 pathway. Lipid peroxidation, driven by free radicals, primarily affects unsaturated fatty acids in cell membranes. This reaction generates lipid peroxides, which break down into toxic by-products like MDA, a major toxic derivative. The imbalance between oxidative damage and the inability to defend against it ultimately leads to ferroptosis [7]. In our study, severe hypothermia induced iron accumulation in cortical neurons, and the upregulation of ACSL4 increased the cells’ sensitivity to ferroptosis. Additionally, a reduction in the levels of Nrf2, SLC7A11, GSH, and GPX4 was observed, resulting in the failure of the antioxidant system and an increase in lipid peroxidation products such as MDA and 4-HNE in the cerebral cortex. Hypothermia-induced ferroptosis has previously been demonstrated in other organs like the myocardium and kidneys, which aligns with our findings. Overall, our results suggest that iron accumulation and the downregulation of the SLC7A11/GSH/GPX4 antioxidant system activate lipid peroxidation, leading to ferroptosis in the cerebral cortex during severe hypothermia.

Fer-1, a potent and specific ferroptosis inhibitor, has been shown to prevent cell death in various models of neuronal diseases, including acute brain contusion, Huntington’s disease, acute brain injury, periventricular leukomalacia, and renal failure. In this study, Fer-1 effectively reduced iron accumulation, increased tolerance to ferroptosis, enhanced the capacity of the antioxidant system, and reduced the formation of lipid peroxidation products. These findings not only clarify the mechanisms underlying cognitive impairment in mice surviving severe hypothermia but also provide an experimental basis for potential clinical treatments targeting ferroptosis and future research directions. At the same time, Fer-1 pharmacokinetic studies and clinical translation related studies still need to be further studied. Whether the Nrf2/SLC7A11/GSH/GPX4 signaling axis is the key pathway leading to ferroptosis in neurons caused by low temperature and whether it can be used as a potential target for the treatment of cognitive dysfunction caused by low temperature still needs further research.

This study extends previous investigations into the mechanisms underlying severe hypothermic injury, demonstrating that ferroptosis contributes to cognitive impairment in mice surviving severe hypothermia. However, the precise pathways through which hypothermia triggers ferroptosis via metabolic disturbances, and the detailed regulatory mechanisms involved, remain unclear. Further research is required to elucidate these processes.

## 4. Materials and Methods

### 4.1. Experimental Animals

All animal experiments were approved by the Experimental Animal Management Committee of Hebei Medical University (approval number: 2022132). A total of 139 male C57BL/6N mice, aged 8 weeks and weighing 25 ± 2 g, were purchased from Beijing Vital River Laboratory Animal Technology Co., Ltd. (Beijing, China). The mice were housed under specific pathogen-free (SPF) conditions, at a constant temperature of 23 ± 2 °C and 50% humidity with a 12 h light/dark cycle. Euthanasia was performed by decapitation following anesthesia with 2% sodium pentobarbital.

### 4.2. Model Preparation and Sample Extraction

The mice were randomly divided into control (CON) and severe hypothermia survival groups (HP1d, HP7d, HP14d, HP21d). Under isoflurane anesthesia, the mice had their fur shaved, particularly around the head, and were then exposed to severe hypothermia by placing them in a 2–6 °C box with continuous ventilation for 90 min [5]. The mice showed signs of coma such as body curling, loss of righting reflex, inability to move due to pricking pain, etc. After this exposure, the mice were removed and rewarmed at room temperature. During rewarming, probably due to physical differences, 11 mice died from severe hypothermia, and the surviving mice were raised under normal conditions.

To investigate the role of ferroptosis in cognitive impairment in mice surviving severe hypothermia, the mice were randomly assigned to four groups: CON, Fer-1, HP14d, and HP14d + Fer-1. The HP14d + Fer-1 group received intraperitoneal injections of Fer-1 (5 mg/kg) [38,39] one hour after rewarming, followed by daily injections of the same dose at 9 a.m. for subsequent days. The Fer-1 group, without a low-temperature process, received the same dosage and injection regimen.

**Figure 8:**
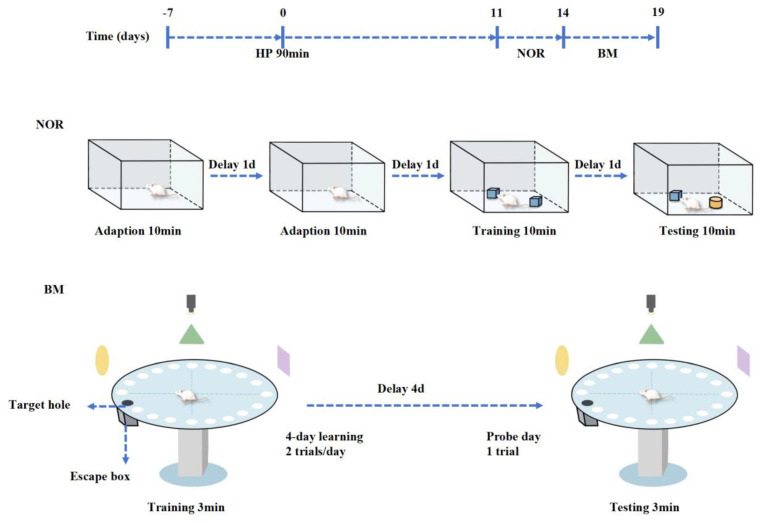
Schematic diagram of behavioral experiment process. After 11 days of normal feeding following severe hypothermia, the HP14d group underwent the NOR and BM experiments. At the end of the experiment, brain tissue was extracted for evaluation of ferroptosis levels, and histological staining was performed on the cerebral cortex.

After the completion of behavioral testing (Figure 8), mice from each experimental group were euthanized by decapitation under anesthesia. Given the critical role of the cerebral cortex in cognitive functions, tissue samples from this brain region were collected for further research.

### 4.3. Behavioral Test

#### 4.3.1. Novel Object Recognition

The NOR is a classic behavioral test to assess recognition memory and cognitive abilities in mice through exposure to novel objects in the environment [40,41]. The experiment is divided into three phases: the adaptation phase, the training phase, and the testing phase. The first two days serve as the adaptation phase, during which the mice are placed in the novel object recognition arena and allowed to explore freely for 10 min to acclimate to the environment. On the third day, the training phase begins, where two identical objects are positioned symmetrically within the arena, placed 10 cm away from the walls. The mice are then allowed to explore the objects freely for 10 min. On the fourth day, during the testing phase, one of the identical objects is replaced with a novel object, and the time spent exploring the new and old objects is recorded over a 10 min period. After the experiment, the mice’s excrement is promptly cleaned, and the arena is wiped with 75% ethanol to prevent odor interference. Each behavioral experiment was conducted at 9 a.m. All data were automatically recorded and analyzed by EthoVision XT15 software (Noldus Informaton Tecnnology bV, Wageningen, The Netherlands). Data on mobility problems due to illness or injury were excluded. The Discrimination Index (DI) is used to evaluate the mice’s learning and memory, calculated as DI = N/(N + F) × 100%, where N represents the time spent exploring the novel object and F represents the time spent exploring the familiar object.

#### 4.3.2. Barnes Maze

The BM is a widely used experimental paradigm to assess spatial learning and memory in mice [42,43]. The procedure consists of two stages: (1) Training phase: The Barnes Maze apparatus consists of a circular platform with 20 holes, one of which has a hidden escape box beneath it (the target box). During each training session, the mice are placed at the center of the maze and confined in a plastic bucket for 5 s. The bucket is then removed, and the mice are allowed to explore the maze freely for 3 min or until they enter the target box, which concludes one session. If the mouse does not find the target box within 3 min, then it is manually placed into the box for 15 s. After each training session, the maze and target box are cleaned with 75% ethanol. The training occurs twice daily with a one-hour interval for four consecutive days. The test board is rotated daily, while the target box’s spatial position remains unchanged, preventing the use of olfactory cues. The time taken for each mouse to enter the target box during training is recorded. (2) Testing phase: On the fifth day, the target box is removed, and the mice are allowed to explore the maze for 3 min. The amount of time spent in the target box area is recorded, serving as an indicator of cognitive function and spatial memory. Each behavioral experiment was conducted at 9 a.m. All data were automatically recorded and analyzed using the DigBehv animal behavior analysis system. Data on mobility problems due to illness or injury were excluded and the mice explored the maze for 3 min. The total time spent in the target box area is recorded, serving as an indicator of cognitive function and spatial memory.

### 4.4. HE Staining

Red neurons are indicative of coagulative necrosis resulting from acute ischemia, hypoxia, or other factors. Their morphological features include nuclear shrinkage, cell body shrinkage and deformation, disappearance of cytoplasmic Nissl bodies, and a dark red cytoplasm when stained with hematoxylin and eosin (HE), a hallmark of red neurons. As necrosis progresses, the nuclei dissolve and disappear, leaving a cell with only a residual outline or trace, referred to as a ghost cell. Brain tissue was fixed, dehydrated, and embedded in paraffin wax, which was then sectioned and dewaxed. HE staining was performed according to standard protocols. Six rats from each group were used for morphological observation and data analysis. Sections were examined and photographed using an optical microscope (BX5; Olympus, Tokyo, Japan), and the average number of red neurons in the cerebral cortex was counted by an observer blinded to the experimental conditions at 400× magnification.

### 4.5. Thionin Staining

Thionin, a basic dye, is widely used for the histological staining of degenerating and dying neurons in the central nervous system [44]. The experimental procedure followed the manufacturer’s instructions (G1438, Solarbio, Beijing, China). Thionin staining was used to visualize neuronal degeneration and death in the cerebral cortex. The average number of Thionin-positive neurons in the cerebral cortex was counted by an observer blinded to the experimental conditions under an optical microscope (BX5; Olympus) at 400× magnification.

### 4.6. Western Blotting Analysis

A mammalian protein extraction kit (Pierce, Thermo Fisher Scientific, Rockford, IL, USA) was used to extract proteins from cerebral cortical tissue. Protein concentration was measured using a BCA protein assay kit. Under reducing conditions, 50 µg of protein was separated by SDS-PAGE and transferred electrophoretically to polyvinylidene fluoride (PVDF) membranes. Specific antibodies were used to detect the expression of Nrf2 (80593-1-RR, Proteintech, Wuhan, China), SLC7A11 (ab307601, Abcam, Cambridge, UK), GPX4 (ab125066, Abcam), ACSL4 (ab155282, Abcam), and 4-HNE (ab48506, Abcam). Following incubation with horseradish peroxidase-conjugated secondary antibodies, chemiluminescence detection was used to analyze the optical density of the target bands, with results processed using a gel image analysis system.

### 4.7. Enzyme-Linked Immunosorbent Assay (ELISA)

The cerebral cortex protein was extracted and the MDA (E-BC-K025-M, Elabscience, Houston, TX, USA), GSH (E-BC-K030-M, Elabscience), and LPO (E-BC-K176-M, Elabscience) ELISA kits were used to detect the content of MDA, GSH, and LPO in the cerebral cortex according to the instructions of the corresponding kit.

### 4.8. Iron Determination

The ferrous iron content in cerebral cortex tissue was quantified using a ferrous iron assay kit (MAK025, Sigma-Aldrich, St. Louis, MO, USA) following the manufacturer’s guidelines. The cerebral cortex tissue was homogenized, and the tissue supernatant was extracted. The supernatant was then mixed with the iron-developing solution as per the instructions, and the levels were detected using an enzyme-labeled instrument (Spectra Max i450x; Molecular Devices, Sunnyvale, CA, USA). Additionally, protein concentrations in cerebral cortex tissue were measured using a BCA kit (23227, Thermo Fisher Scientific), and ferrous iron levels were normalized to protein concentrations.

### 4.9. Statistical Analysis

In the morphological experiment, two independent observers counted the number of positive cells in the cerebral cortex of each mouse, without knowing the experimental conditions. Data analysis was performed using R software (version 4.2.2). Normally distributed data were presented as mean ± standard deviation (SD) from three independent experimental replicates. For comparisons between two groups with normal distribution and homogeneity of variance, Student’s *t*-test was employed, while comparisons among three or more groups were performed using ANOVA followed by Bonferroni multiple comparisons. A *p*-value of less than 0.05 was considered statistically significant.

## 5. Conclusions

Severe hypothermia can lead to cognitive dysfunction and cortical neuronal damage in surviving mice. Ferroptosis mediates the cognitive impairment and cortical neuronal damage caused by severe hypothermia in surviving mice. The targeted inhibition of ferroptosis can effectively improve the cognitive dysfunction and cortical neuronal damage caused by severe hypothermia. Future research will further investigate the specific molecular mechanisms by which hypothermia-induced ferroptosis leads to cognitive dysfunction. These issues should be addressed in subsequent studies.

## Data Availability

The datasets analyzed during the current study are available from the corresponding author upon reasonable request.
